# Using RNA-based therapies to target the kidney in cardiovascular disease

**DOI:** 10.3389/fcvm.2023.1250073

**Published:** 2023-10-06

**Authors:** Trecia C. Palmer, Robert W. Hunter

**Affiliations:** ^1^ Centre for Cardiovascular Science, The Queen's Medical Research Institute, University of Edinburgh, Edinburgh, United Kingdom; ^2^ Department of Renal Medicine, Royal Infirmary of Edinburgh, Edinburgh, United Kingdom

**Keywords:** chronic kidney disease (CKD), cardiovascular disease (CVD), ribonucleic acid (RNA)-based therapy, RNA interference (RNAi), small interfering RNA (siRNA), nanoparticles, nanocarrier

## Abstract

RNA-based therapies are currently used for immunisation against infections and to treat metabolic diseases. They can modulate gene expression in immune cells and hepatocytes, but their use in other cell types has been limited by an inability to selectively target specific tissues. Potential solutions to this targeting problem involve packaging therapeutic RNA molecules into delivery vehicles that are preferentially delivered to cells of interest. In this review, we consider why the kidney is a desirable target for RNA-based therapies in cardiovascular disease and discuss how such therapy could be delivered. Because the kidney plays a central role in maintaining cardiovascular homeostasis, many extant drugs used for preventing cardiovascular disease act predominantly on renal tubular cells. Moreover, kidney disease is a major independent risk factor for cardiovascular disease and a global health problem. Chronic kidney disease is projected to become the fifth leading cause of death by 2040, with around half of affected individuals dying from cardiovascular disease. The most promising strategies for delivering therapeutic RNA selectively to kidney cells make use of synthetic polymers and engineered extracellular vesicles to deliver an RNA cargo. Future research should focus on establishing the safety of these novel delivery platforms in humans, on developing palatable routes of administration and on prioritising the gene targets that are likely to have the biggest impact in cardiovascular disease.

## Introduction

RNA-based therapies are emerging as an effective and versatile class of medications in a range of diseases. So far, they have been largely limited to small interfering RNAs (siRNAs) that mediate gene silencing in hepatocytes and mRNA vaccines. Their full potential will be unlocked when we learn how to target them effectively to other cell types. In this review, we consider recent progress towards developing RNA-based therapies that can selectively target kidney cells and in particular renal tubular epithelial cells (rTECs). Given the central role of the renal tubules in maintaining cardiovascular homeostasis, such novel therapies could have the greatest impact in treating cardiovascular disease (CVD).

We begin by briefly introducing the concepts of RNA-based therapy and reviewing the causative relationship between kidney disease and CVD. We then discuss attempts to deliver RNA-based therapies selectively to rTECs in animal models and humans. We conclude by positing the potential clinical impact that these novel therapeutics could have in cardiovascular disease.

## The advantages of RNA-based drugs

### How RNA-based drugs work

There are several different classes of RNA therapeutic, including mRNAs, siRNAs, RNA aptamers and CRISPR guide RNAs (1). In principle, any class of coding or non-coding RNA could form the basis of a drug (2), as could novel engineered RNAs such as self-amplifying RNAs (3). However, three strategies are currently most advanced in terms of their clinical development.

In the first, siRNA, shRNA (short hairpin RNA) or anti-sense oligonucleotides (ASOs) silence the expression of a pathogenic mRNA through RNA interference (RNAi). These molecules directly bind their cognate mRNA and inhibit protein synthesis. This process is the mammalian correlate of a phenomenon that was first characterised in plants and *C elegans* (4). In the second, ASOs are used to inhibit microRNAs (miRNAs): the endogenous negative regulators of mRNA expression. Such “antagomirs” thus de-repress expression of beneficial mRNAs. In the third major class, mRNA molecules increase the expression of a gene of interest (5). This can be a successful strategy for immunisation, as the Moderna Covid-19 vaccine powerfully demonstrated (6). It could also restore expression of deficient protein or mediate gene editing in a range of diseases.

### Why RNA-based drugs offer advantages over conventional small molecule drugs

Our current approach to treating kidney and cardiovascular disease relies almost entirely on conventional small molecule drugs. These drugs have been developed over decades, often more by accident than design. They tend to have rapid metabolism—and so require daily or twice-daily dosing—and to target receptors that have been chosen predominantly because they are easily “druggable”, rather than because they are necessarily the most important points in a disease pathway. Drug development is slow, in part because of the relatively unpredictable safety profile of small molecules.

RNA-based therapies could potentially solve all of these problems (7, 8). RNA-based therapies can be designed to target, in principle, any gene of interest. Defined therapeutic classes, such as siRNA, have standard pipelines for development and manufacture, allowing for rapid, relatively inexpensive drug development. They also have predictable pharmacokinetic properties. Fortuitously, liver-targeting siRNAs appear to induce long-lasting therapeutic gene silencing (9, 10), so that dosing might be required every few months, potentially improving drug adherence.

However, for the successful clinical application of RNA-based therapies in kidney disease, we must first learn how to direct these drugs selectively to kidney cells. After intravenous injection, naked RNA is widely distributed amongst different body tissues (11). There are also additional challenges: ensuring biological safety, determining the most effective route of administration and overcoming off-target effects.

## The kidney is a therapeutic target in cardiovascular disease

### The kidneys maintain cardiovascular homeostasis

The kidneys maintain cardiovascular homeostasis by regulating the circulating volume and blood pressure (12). Therefore, many extant drugs used for preventing or treating cardiovascular disease target the kidney and specifically the most abundant kidney cell, the renal tubular epithelial cell (rTEC). Renin-angiotensin system inhibitors, sodium-glucose co-transporter inhibitors (SGLT2i), diuretics and mineralocorticoid inhibitors, which have all been shown to reduce cardiovascular disease in various clinical contexts, act predominantly by altering kidney solute transport (13). Therefore, the concept of targeting the kidney to treat or prevent cardiovascular disease is well-established.

### Kidney disease causes cardiovascular disease

The other rationale for targeting the kidney to treat cardiovascular disease, is that chronic kidney disease (CKD) is a leading risk factor for cardiovascular disease. Therefore, treating kidney disease *per se* will reduce cardiovascular disease.

CKD is the eighth leading cause of death worldwide (14), with just over 10% of the adult population affected to some degree (13, 15). It is associated with excess morbidity and mortality, an association that is not fully explained by confounding from co-morbid cardiovascular disease (CVD), diabetes and hypertension (16). The leading cause of death in CKD is cardiovascular disease (CVD), accounting for 40%–50% of deaths (17), compared to ∼33% of deaths in the general population (18). Both GFR and albuminuria are independent risk factors for mortality and myocardial infarction (19). The mechanisms whereby loss of glomerular filtration (GFR) or albuminuria cause cardiovascular disease are still relatively poorly understood, but likely involve volume expansion, increases in cardiac pre-load and after-load, perturbed calcium-phosphate homeostasis and increased exposure to circulating “uraemic” metabolites (20).

### How could RNA therapies treat kidney and cardiovascular disease?

RNA-based therapies are already being used in the clinic to treat kidney disease, but not by directly targeting kidney cells. For example, Lumasiran is an siRNA that targets glycolate oxidase in hepatocytes that is effective at treating primary hyperoxaluria (21). Similarly, RNA-based therapies for cardiovascular disease target gene expression in the liver: angiotensinogen for hypertension, or PCSK9 or apolipoprotein B-100 for hypercholesterolaemia (22–24). Underscoring the tight association between kidney and cardiovascular health, liver-targeting angiotensinogen siRNA protected against kidney damage in a rat subtotal nephrectomy model of CKD (25).

There are some other metabolic diseases in which targeting gene expression in the liver will improve kidney outcomes, but for the vast majority of kidney diseases, it will be necessary to develop therapies that can directly regulate gene expression in kidney cells. The capacity for treating cardiovascular disease will be hugely augmented if we can develop drugs that target the kidneys, given their central role in cardiovascular homeostasis. Therefore, potential strategies for kidney-specific RNA delivery are being tested extensively in pre-clinical models. These are summarised in Table 1 and we chose some interesting examples to discuss in the text below.

**Table 1:** Kidney-targeting siRNA-based therapies in pre-clinical and clinical development.

Pre-clinical trials	Reference	Cell/tissue type targeted	Disease model	Carrier and route of administration
siRNA target				
Trp53	Molitoris et al. (26)	Proximal tubular epithelial cells	Cisplatin-induced and ischemic AKI models in rats	Naked; IV
Egfp and Tgfb1	Takabatake et al. (27)	Glomeruli	Glomerulonephritis model in rats	Naked, renal artery
Mapk1	Shimizu et al. (28)	Glomeruli	Glomerulonephritis model in mice and rats	Polyion complex nanocarriers; IP
Smad4	Morishita et al. (29)	Tubulointerstitium and tubule Epithelial cells	Renal fibrosis model in mice	Naked; IV
Cox2	Yang et al. (30)	Peritoneal macrophages recruited to the kidney	UUO model in mice	Chitosan NPs; IP
Fas, C3 and RelB	Zheng et al. (31)	Glomeruli and medullar tubule cells	Ischemic AKI in mice	Naked; renal artery
Ctr1, Trp53 and Mep1b	Alidori et al. (32)	Cortex and PTECs	AKI model in mice	Fibrillar carbon nanotubes (fCNT); IV
Cd40	Narváez et al. (33)	Tubulointerstitium	UUO model in mice	Naked; IV
p38a MapK and p65	Wang et al. (34)	Glomerular mesangium and peritubular endothelial cells	Glomerulonephritis model in mice	Liposomal NPs, IV
Trp53	Thai et al. (35)	Tubular epithelial cells	AKI model in mice	DNA nanostructure; IV
Caspase-3 and complement-3 siRNA	Zheng et al. (36)	Proximal tubular epithelial cells	Renal ischaemia reperfusion injury	Naked; IV
HSP47	Xia et al. (37)	Proximal tubular epithelial cells	Tubulointerstitial fibrosis	Gelatine microspheres; intraurethral
P53	Thai et al. (35)	Proximal tubular epithelial cells	AKI (polymicrobial-induced)	DNA tetrahedron nanovehicle; IV
miR-21	Gomez et al. (38), Rubel et al. (39)	Tubular epitheilial cells and podocytes	Alport nephropathy	Naked, SC
miR-17	Lee et al. (10)	Tubular epithelial cells	Autosomal dominant polycystic kidney disease	Naked, SC
Clinical trial/FDA approved	Reference	Tissue type targeted	Kidney disease	Carrier, administration and status
siRNA target				
Glycolate oxidase	Liebow et al. (40, 41)	Liver	Oxaluria	GalNAc; Sc; FDA Approved on 11/20/2019 (Lumasiran)
Lactate dehydrogenase	Lai et al. (41, 42)	Liver	Oxaluria	GALNAc; SC; Phase 3 Clinical trial (Nedosiran/NCT04042402)
p53	Thompson et al. (41, 43)	Proximal tubule	Delayed graft function and acute kidney injury after cardiac surgery	Naked; IV; Phase 3 Clinical trial (Teprasiran (NCT02610296)
QPI-1002	Thielmann et al. (44)	Proximal tubule	ARF, AKI delayed graft function post transplantation	Phase I- NCT00554359 Phases I/II- NCT00802347

Trp53, transformation related protein 53; Egfp, epidermal growth factor protein; Tgfb1, transforming growth factor beta 1; Mapk1, mitogen-activated protein kinase 1; Smad4, suppressor of mothers against decapentaplegic homolog 4; Cox2, cyclooxygenase-2; C3, complement factor 3; RelB, reticuloendotheliosis viral oncogene homolog B; Ctr1, copper transporter 1; Mep1b, meprin A subunit beta; CD40, cluster of differentiation 40; p38a MapK, p38a mitogen-activated protein kinase; p65, transcription factor p65/nuclear factor NF-kappa-B p65 subunit; HSP47, heat shock protein 47; P53, p53 tumour suppressor gene; QPI-1002, teprasiran (Quark Pharmaceuticals); IV intravenous; IP, intraperitoneal; SC, subcutaneous; AKI, acute kidney injury; UUO, unilateral ureteral obstruction.

## How can we target kidney cells with siRNA?

### The challenge

Naked RNA is a negatively-charged macromolecule that does not easily cross the hydrophobic, negatively-charged cell membrane. In the absence of modifications, naked RNA is subject to nuclease degradation and clearance by the kidneys and reticuloendothelial system (45, 46). The challenge is to learn how we can modify RNA molecules, or package them within delivery vehicles, so as to enhance delivery to kidney cells. An ideal delivery vehicle would selectively deliver its RNA cargo to rTECs and preserve the functional effects of the RNA in recipient cells (e.g., gene silencing). It should be able to deliver RNA across the cell membrane and not into endosomal degradation pathways. It should also be non-immunogenic, non-toxic, biologically inert, cheap to manufacture and delivered through a patient-friendly route (e.g., subcutaneous injection) (47).

**Figure 1:**
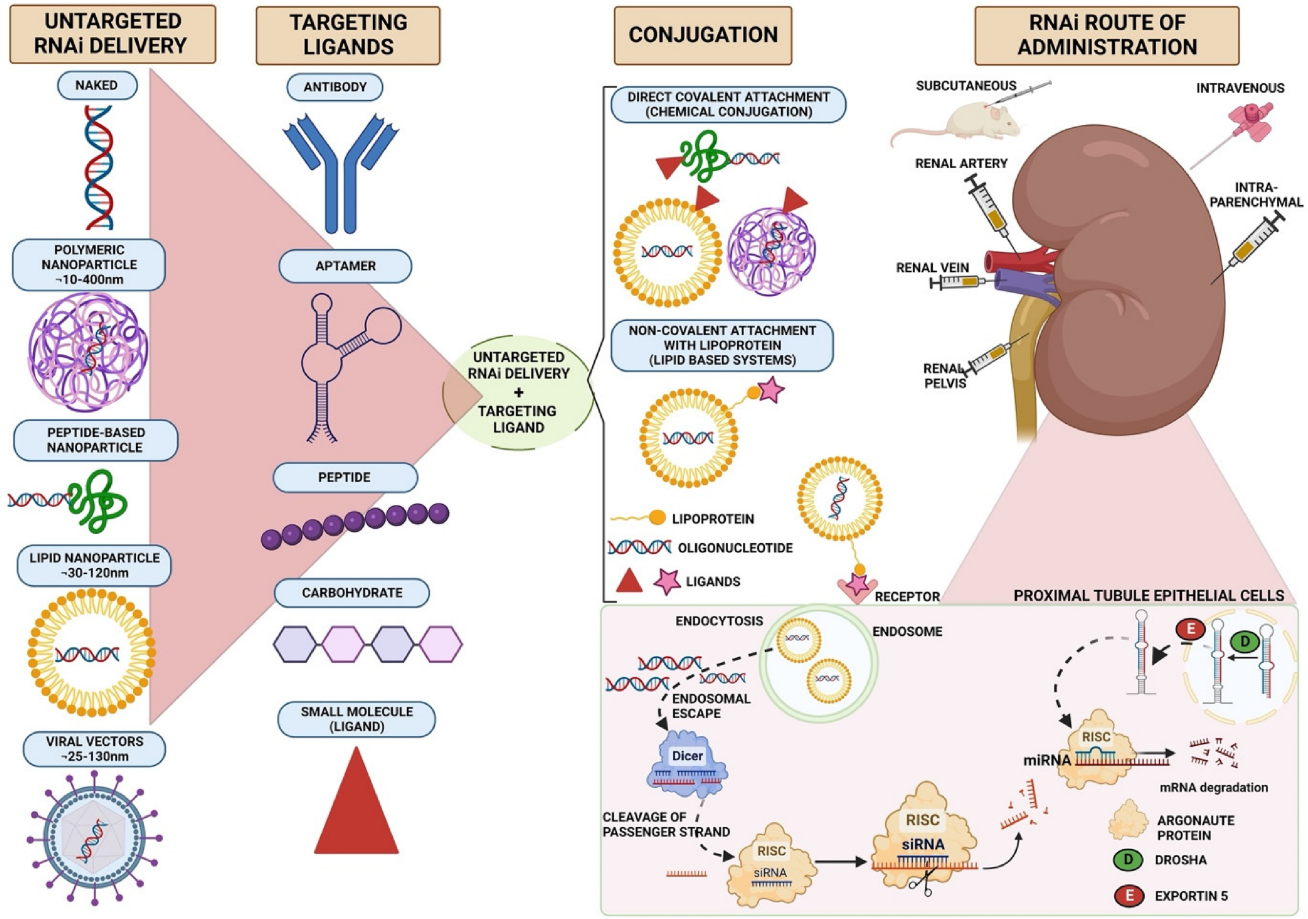
Strategies for selective delivery of siRNA to kidney cells. siRNA can be packaged into lipid-based, polymer-based, viral-based systems or extracellular vesicles. Viral vectors or nanoparticles conjugated to different targeting ligands, such as antibodies, aptamers, peptides or small molecules, form different carrier systems that are preferentially internalised by kidney cells. These targeting delivery vehicles bind to their cognate receptor on kidney cells and are endocytosed. Exogenous siRNA enters the cytosol by endosomal escape. siRNA is then cleaved by dicer enzyme into a passenger strand, which is recycled, and the guide strand loaded onto the RISC complex. siRNA degrades mRNA strand by full complementarity and inhibits protein synthesis. Diagram created in Biorender.

Broadly, RNA can be delivered as “naked” nucleic acid or in delivery systems that are classified as viral or non-viral (Figure 1).

### Naked RNA

Naked nucleic acids, injected systemically, are excreted in the urine and accumulate within the kidney, particularly in renal tubular cells (48–51). This kidney delivery is not selective; naked RNA is also delivered to liver, spleen, lung, heart and adipose tissue (11, 48, 49, 52, 53). Nor is naked RNA very stable: the systemic half-life of unmodified siRNA is measured in seconds to minutes (11, 53).

The stability and biodistribution of siRNA can be altered by chemical modification of the RNA backbone. Phosphorothioate siRNA accumulates preferentially in the kidneys, relative to other organs (11). Phosphorothioate modification enhances uptake from the peritubular capillaries in isolated perfused rat kidneys (54). There is also uptake from the urinary space. This might, in part, be mediated through endosomal receptors in proximal rTECs; in monkeys, 2′-O-methyl phosphorothioate oligonucleotides appear to compete with low-molecular proteins for uptake into rTECs (55).

In contexts where rapid clearance and off-target RNA delivery is not problematic, naked RNA could be used to therapeutic effect. Naked siRNA targeting the tumour suppressor gene p53 has been tested in pre-clinical and clinical trials of acute kidney injury. Intravenous administration gave effective delivery to rTECs and prevented kidney injury in rat models of ischemia-reperfusion injury and cisplatin toxicity (26). This siRNA is now in phase three clinical trials for the treatment of delayed graft function after transplantation (NCT02610296), and acute kidney injury after cardiac surgery (NCT03510897) (43).

Some oligonucleotides appear to be selectively delivered to kidney cells when delivered in their naked form. For example, an ASO directed against miRNA-17 performed well in pre-clinical trials of polycystic kidney disease (ADPKD) (10). Intriguingly, this oligonucleotide was preferentially delivered to the kidney as opposed to the liver, and specifically delivered to renal tubular epithelial cells. The mechanism directing this specific delivery is unknown, although the authors of that study speculate that it may be related to the short length of the ASO (9-nucleotides). This is now being tested in a phase I human trial (NCT04536688). Similarly, the miR-21 antagomir, Lademirsen, accumulates in kidney cells and was effective in mouse models of Alport nephropathy (38, 39). However, a phase 2 clinical trial was terminated after a futility analysis (NCT02855268), highlighting the need for careful selection of candidate oligonucleotides for clinical testing and appropriate trial design—as for any other drug class.

### Viral systems

Viral vectors are highly effective at delivering genetic material to target cells (56). Viral vectors can be chosen based on an inherent tropism for a target cell type or tissue or, engineered to enhance this tropism. For example, delivery of shRNA to kidney cells has been achieved using AAV2 in rats (57). Viral vectors may be inactivated by removing the viral genome to generate a non-infectious delivery vector, circumventing the safety concerns of live virus. One such envelope vector, derived from the haemagglutinating virus of Japan (HVJ) delivered cDNA for a mitochondrial membrane protein to glomerular and renal tubular cells in diabetic mice (58).

Whilst the risks of cellular toxicity and immune reactions are minimised with third generation adenoviral vectors (59), there is a concerted effort to develop synthetic carriers for RNA such as nanoparticles or extracellular vesicles. These could eliminate the safety concerns of viral vectors and offer advantages in terms of cheap, scalable manufacturing and flexibility in design.

### Lipid-based nanoparticles

Lipid-based nanoparticles (LNPs) are the most established non-viral RNA delivery system (60–63). These are typically <100 nm in diameter, can be engineered to traverse the phospholipid cell membrane and have very little toxicity and immunogenicity (63).

One issue that has faced investigators developing LNPs to target organs other than the kidney, is that renal elimination is the major route of LNP clearance. Therefore LNPs are typically modified, e.g., by including PEG-lipids, to *minimise* renal clearance (63). This might of course be a counterproductive strategy in the development of any kidney-targeting therapy. Indeed, there are very few examples of LNPs that have been developed to deliver RNA to kidney cells, and certainly to rTECs. As proof of principle that this could be possible, one group were able to effect gene silencing in mesangial and glomerular endothelial cells in a mouse model of IgA nephropathy, using polyethyleneimine-containing, octa-arginine-coated liposomes (34).

### Polymer-based nanoparticles

Synthetic polymers have been used to stabilise and deliver RNA (64). Negatively charged nucleic acids coupled with positively charged polymers to form polyplexes (60). Polymers that have been employed include polyethylenimine (PEI), poly-(ethylene-glycol)-poly-(L-lysine) (PEG-PLL), poly (lactide-co-glycolide) (PLGA), poly-amidoamine dendrimers (PAMAMs) and the fungal polysaccharide chitosan (47, 65).

Many polymer particles are sufficiently small (<20 nm) that they ought to cross the glomerular filtration barrier. In mice, a PEG-PLL carrier significantly enhanced delivery of fluorescently-tagged siRNA to kidneys in mice, compared to a naked siRNA control (28). A MAPK-targeting siRNA delivered through this system mediated gene silencing in glomerular cells and exerted a therapeutic effect in a glomerulonephritis model.

Chitosan particles showed promise as a kidney-targeting strategy in an early murine biodistribution study, with siRNAs accumulating in the kidney, 24 h after intravenous administration (11). The likely mechanism of this effect was subsequently demonstrated using an elegant transgenic model. AQP1-targeting siRNA delivery was restricted to megalin-expressing rTECs in a mosaic megalin knockout mouse, providing compelling evidence that these nanoparticles cross the glomerular filtration barrier and are then internalised by the multi-ligand endocytic receptor megalin (66).

Branched, symmetrical “dendrimers” are a polymer subclass with particularly attractive properties with respect to RNA delivery (67). They have multiple surface groups to which targeting ligands could be attached. For example, dendrimers decorated with folic acid or the small cyclo peptide cRGDfC have been used to deliver siRNA selective to proximal tubular cells (expressing the folic acid receptor) or podocytes (expressing α_v_β_3_ integrin) respectively in murine kidney disease models (68, 69).

Recent developments in kidney-targeting RNA delivery vehicles have involved nanoparticles with sophisticated molecular structures. In mice, modified carbon nanotubules have been used to deliver siRNA into rTECs: a strategy that induced therapeutic effects when silencing p53 and the metalloprotease Meprin-1β in cisplatin-induced AKI (32). The authors of that study speculate that carbon nanotubes are able to traverse the glomerular filtration barrier by virtue of being long and thin, whereas more globular nanoparticles tend to accumulate in the liver. Nanosized tetrahedra, constructed from DNA (10 bp per side), were able to deliver siRNA specifically into rTECs and again conferred protection from cisplatin injury via p53 silencing (35). Although these strategies clearly show great promise, as selective delivery relies on the physiochemical properties of the delivery vehicle—rather than recognition of a specific ligand by a cell-surface receptor—they may be limited in their ability to target specific rTEC subtypes.

### Extracellular vesicles

Extracellular vesicles are released by all cells into the extracellular space. They are either derived from the plasma membrane or the endosomal pathway (“exosomes”) and carry with them a subset of cellular macromolecules, including RNA (47, 70, 71). Their cellular origin endows them with inherently low toxicity and immunogenicity. They may also offer a natural solution to the targeting problem as they are preferentially internalised by cells of the same type from which they were derived (72).

Furthermore, EVs can be readily engineered to express surface receptors to enhance delivery to specific target cells. For example, EVs engineered to express the rabies virus glycoprotein (RGV) peptide gave enhanced delivery of an siRNA cargo to kidney cells (73, 74), because kidney cells express the RGV ligand, the nicotinic acetyl choline receptor (75). Similarly, EV delivery can be directed by modulating surface immunoglobulin (76) or integrin expression (77).

One elegant demonstration of the power and flexibility of this approach was provided by an experiment designed to deliver siRNA specifically to *diseased* rTECs. When erythrocyte-derived vesicles were engineered to express a KIM-1 ligand, they delivered cargo siRNA preferentially to injured rTECs in murine ischaemia-reperfusion and ureteral obstruction models (78). Delivery of siRNAs targeting p65 and Snail ameliorated tubulointerstitial fibrosis. The ability to target a defined subset of tubular epithelial cells (or indeed other kidney cell types) would open up a range of subtle therapeutic options with minimal off-target side-effects. For example, depletion of senescent rTECs has been shown to improve recovery from ischaemia-reperfusion injury in a mouse model (79); could a selective senolytic therapy be achieved by delivering pro-apoptotic RNAs selective to senescent rTECs?

As RNA carriers can be engineered to express surface ligands that direct them to kidney cells, the obvious question is: which ligands will deliver these vehicles most effectively to kidney target cells (e.g., rTECs)? A variety of ligand classes have been tested: peptides, antibodies, aptamers, carbohydrates, and small molecules (Figure 1). Ligands for the major proximal rTEC receptors megalin, cubulin and transferrin are obvious candidates (and we have already discussed some above). Antibodies or antibody-like molecules have been used to target kidney endothelial cells (anti-VCAM1) and proximal tubular cells (anti-CD11b and anti-CD163); however, their use may be limited by their large size which leads to deposition of immune complexes on the glomerular basement membrane and therefore a risk of glomerulonephritis (60, 80–85).

## Administration routes for kidney-targeting RNA therapies

Nanoparticles delivered into the systemic circulation face significant hurdles to reaching the kidney. They may be degraded by the reticuloendothelial system or—depending on their chemical composition—be preferentially delivered to the liver. Furthermore, there is uncertainty as to the extent to which nanoparticles can cross an intact glomerular filtration barrier. Some provocative studies suggest that fluorescently-labelled extracellular vesicles can traverse this barrier in healthy mice and humans, even though their size and charge profile ought to be prohibitive (86, 87).

In pre-clinical models, direct administration into the renal artery, renal vein, ureter or kidney parenchyma have been used to deliver RNA to the kidney, but the translational potential of these routes is obviously limited (37, 60). One intriguing research question is whether physical methods could be used to enhance selective delivery of a systemically-administered RNA drug. It may be possible to modify delivery of RNA vehicles using ultrasound, electromagnetic stimuli or light (60, 88). For example, after systemic delivery of the drug delivery vehicle, focussed ultrasound beams can direct the vehicle to specific cell types and, via acoustic cavitation, induce localised cargo release. This approach was successful for enhancing kidney delivery of a DNA plasmid in mice (89). Alternatively, electroporation of the kidney also enhanced delivery of siRNA and shRNA in mice, after intra-arterial administration (27, 90).

## Conclusions and perspectives

RNA-based therapies offer a powerful opportunity to target new pathways in cardiovascular disease. Existing RNA-based therapies largely target gene expression in hepatocytes and are therefore limited in their ability to treat cardiovascular disease. The kidney is an attractive target in cardiovascular disease because the kidney plays a central role in maintaining cardiovascular homeostasis and because CKD is a leading risk factor for cardiovascular disease.

Various approaches to delivering RNA selectively to kidney cells are being tested in pre-clinical models. Polymer and vesicle-based delivery systems show promise, particularly in their ability to deliver siRNA selectively to rTECs, without inciting toxicity or immune activation. Extracellular vesicles can be engineered to express surface ligands that interact with receptors on specific kidney cells, permitting highly selective delivery of RNA to target cells. Several lines of evidence suggest that megalin (and perhaps also cubulin) mediates uptake of exogenous RNA from the tubular space, whether the RNA is delivered as naked nucleic acid or complexed to polymeric delivery vehicles. Delineating the precise molecular details of this pathway and its relative contribution to total kidney RNA uptake will be important areas of study.

Future research should also focus on establishing the safety of these novel delivery platforms in humans and on developing palatable routes of administration. In particular, strategies that induce long-lasting gene silencing could offer advances in drug adherence, which would help to reduce the prevalence of cardiovascular disease. Therefore, the potential targets of novel RNA-based therapies include established targets of conventional small-molecule drugs (e.g., renin-angiotensin and SGLT2 inhibitors) as well as a theoretically unlimited array of novel targets.
